# Resection of Cavernous Malformations

**DOI:** 10.3171/2019.7.FocusVid.Intro

**Published:** 2019-07-01

**Authors:** Jacques J. Morcos, Gary K. Steinberg, Wouter I. Schievink, Georgios A. Zenonos

**Affiliations:** 1 Department of Neurological Surgery, University of Miami, Miami, Florida;; 2 Department of Neurosurgery, Stanford University School of Medicine, Stanford, California; and; 3 Department of Neurosurgery, Cedars-Sinai Medical Center, Los Angeles, California

It is our great honor to have been chosen to co-edit this landmark publication of the inaugural issue of *Neurosurgical Focus: Video*. The Editor-in-Chief could not have chosen a more appropriate topic to herald this video-based journal: resection of cavernous malformations.

The reason the topic is most appropriate for this first issue is because cavernous malformations are one of the few truly microsurgical diseases, not amenable to medical, endovascular, radiation therapy, or even (most would agree) radiosurgical treatment. They also represent a surgical paradox. On the one hand, they are conceptually a rather simple pathological entity: a mixture of blood-filled “caverns” surrounded by a wall of hemosiderin, occult on angiography, with a tendency to grow or hemorrhage in a centrifugal pattern. In other words, they are an ideal surgical target, and a rather easy one at that. They do not bleed very much, and for the most part, they are quite distinct from the surrounding brain or spinal cord tissue. The surgical goal is crisp, clear, and self-evident: remove the lesion, the entire lesion, and nothing but the lesion. Their simplicity in structure is very seductive to the eager surgeon. Their resection is hardly ever accompanied by any of the drama and turmoil that can characterize the clipping of a giant aneurysm, the resection of a complex arteriovenous malformation, or the time-sensitive construction of an interposition high-flow bypass.

On the other hand, this rather innocuous target is a master of deceit. Cavernomas arise anywhere in gray matter, white matter, cranial nerves, or spinal cord. Their locations span the entire spectrum of functional eloquence, surgical depth, and accessibility. Their etiology remains a mystery in spite of a very strong genetic basis. Why would these clearly acquired lesions be associated with a clearly congenital lesion—venous angiomas? Why are they often intimately attached to venous angiomas, and other times quite distant from them? How can the surgeon avoid leaving residual cavernoma, in his or her effort to preserve the angioma? Why do they sometimes recur after a seemingly complete resection?

But the reason a cavernous angioma represents the quintessential formative ground for every neurosurgeon, young or old, is not because of its texture or shape or even size. It is not because it may have bled before and caused a deficit or simply presented with seizures. The “signature” of a cavernous angioma is—like real estate—its location. The 1-cm mulberry, in the frontal pole, is indeed the surgical domain of a well-supervised delighted junior neurosurgical trainee. Yet, the same mulberry in the midst of the pons will be tackled by only a handful of the world’s experts. In other words, once a cavernoma has been reached surgically, there is limited variability in how it is resected: empty the hematoma; surround the lesion; stay within the gliotic plane if in highly eloquent brain, or remove the gliotic plane if in ineloquent terrain and worried about seizures; preserve a large venous angioma; and finally, inspect the cavity for completeness of the resection. The difficulty and challenge in cavernoma surgery is in designing the right approach. It may not be apparent in lesions presenting on surfaces, but choosing the correct surgical trajectory is the entire operation for deep-seated lesions, and in the brainstem, a 1-mm depth is plenty deep.

So what is the secret recipe in designing the right approach? Certainly, the fiber tract and nuclei anatomy is a good starting point. However, no one ever—hopefully—operates on a normal brain, and cavernomas distort the normal anatomy. To account for distortion, high-fidelity and high-resolution imaging, often coupled with functional imaging, has been of great value in helping the surgeon select the surgical path of least destruction. Intraoperative navigation allows fine-tuning of the surgical approach, while intraoperative neurophysiological interrogation of the relevant neural substrate will ensure the absence of ongoing harm to normal structures. After all, no two patients are the same. In essence, the cavernous angioma surgeon needs to be a “complete” surgeon: skilled in microsurgery, with intimate knowledge of normal and distorted anatomy at an advanced level, and with enough ingenuity to create or modify surgical trajectories as dictated by the infinite variations on the interplay between cavernoma and surrounding structures.

After announcing the theme of this issue, we were immediately inundated with high-quality video submissions. Unfortunately, we only had capacity for 30 videos and regrettably had to turn away great work. Although the brainstem makes up less than 10% of the brain, more than two-thirds of the submissions were about brainstem cavernous angiomas. Not surprisingly, and in spite of great strides toward a better understanding of brainstem anatomy, physiology, and “safe entry zones,” surgery of the brainstem still captures the attention and imagination of the skilled microsurgeon more than any other part.

We are fortunate to be including in this July issue representative work from all corners of the globe, and from centers of excellence. The authors are highly skilled operators and the narrated videos are of exceptional quality. The chosen cavernomas span the entire central neuraxis. The reader of this entire July video issue will undoubtedly be enriched with newfound knowledge: a review of cadaveric dissections, a refresher on fiber tract and nuclei anatomy, the subtleties in surgical positioning, technical tips and tricks in approaching and resecting cavernomas, creative ideas regarding novel surgical approaches, evolving principles of intraoperative mapping and monitoring, and much more. Our sincere thanks go to all who contributed to these efforts.

